# Contrast agent-free functional magnetic resonance imaging with matrix pencil decomposition to quantify abnormalities in lung perfusion and ventilation in patients with cystic fibrosis

**DOI:** 10.3389/fmed.2024.1349466

**Published:** 2024-06-05

**Authors:** Felix Doellinger, Grzegorz Bauman, Jobst Roehmel, Mirjam Stahl, Helena Posch, Ingo G. Steffen, Orso Pusterla, Oliver Bieri, Mark O. Wielpütz, Marcus A. Mall

**Affiliations:** ^1^Department of Radiology, Charité-Universitätsmedizin Berlin, Corporate Member of Freie Universität Berlin and Humboldt-Universität zu Berlin, Berlin, Germany; ^2^Division of Radiological Physics, Department of Radiology, University of Basel Hospital, Basel, Switzerland; ^3^Department of Biomedical Engineering, University of Basel, Basel, Switzerland; ^4^Department of Pediatric Respiratory Medicine, Immunology and Critical Care Medicine, Charité-Universitätsmedizin Berlin, Corporate Member of Freie Universität Berlin and Humboldt-Universität zu Berlin, Berlin, Germany; ^5^Berlin Institute of Health at Charité-Universitätsmedizin Berlin, Berlin, Germany; ^6^German Center for Lung Research (DZL), Associated Partner Site, Berlin, Germany; ^7^Department of Pediatric Hematology and Oncology, Charité-Universitätsmedizin Berlin, Corporate Member of Freie Universität Berlin and Humboldt-Universität zu Berlin, Berlin, Germany; ^8^Department of Diagnostic and Interventional Radiology, University Hospital of Heidelberg, Heidelberg, Germany; ^9^Translational Lung Research Center Heidelberg (TLRC), German Center for Lung Research (DZL), Heidelberg, Germany; ^10^Department of Diagnostic and Interventional Radiology with Nuclear Medicine, Thoraxklinik at University Hospital of Heidelberg, Heidelberg, Germany

**Keywords:** magnetic resonance imaging, matrix pencil decomposition, cystic fibrosis, quantitative imaging, functional imaging

## Abstract

**Background:**

Previous studies showed that contrast-enhanced (CE) morpho-functional magnetic resonance imaging (MRI) detects abnormalities in lung morphology and perfusion in patients with cystic fibrosis (CF). Novel matrix pencil decomposition MRI (MP-MRI) enables quantification of lung perfusion and ventilation without intravenous contrast agent administration.

**Objectives:**

To compare MP-MRI with established morpho-functional MRI and spirometry in patients with CF.

**Methods:**

Thirty-nine clinically stable patients with CF (mean age 21.6 ± 10.7 years, range 8–45 years) prospectively underwent morpho-functional MRI including CE perfusion MRI, MP-MRI and spirometry. Two blinded chest radiologists assessed morpho-functional MRI and MP-MRI employing the validated chest MRI score. In addition, MP-MRI data were processed by automated software calculating perfusion defect percentage (QDP) and ventilation defect percentage (VDP).

**Results:**

MP perfusion score and QDP correlated strongly with the CE perfusion score (both *r* = 0.81; *p* < 0.01). MP ventilation score and VDP showed strong inverse correlations with percent predicted FEV1 (*r* = −0.75 and *r* = −0.83; *p* < 0.01). The comparison of visual and automated parameters showed that both MP perfusion score and QDP, and MP ventilation score and VDP were strongly correlated (*r* = 0.74 and *r* = 0.78; both *p* < 0.01). Further, the MP perfusion score and MP ventilation score, as well as QDP and VDP were strongly correlated (*r* = 0.88 and *r* = 0.86; both *p* < 0.01).

**Conclusion:**

MP-MRI detects abnormalities in lung perfusion and ventilation in patients with CF without intravenous or inhaled contrast agent application, and correlates strongly with the well-established CE perfusion MRI score and spirometry. Automated analysis of MP-MRI may serve as quantitative noninvasive outcome measure for diagnostic monitoring and clinical trials.

## Introduction

Cross-sectional imaging enabling the detection and localization of morphological and functional abnormalities of the lungs plays an important role in diagnostic monitoring of patients with cystic fibrosis (CF) (1, 2). The ability to localize and quantify pulmonary changes, ideally by noninvasive and effort-independent investigation, facilitates individualized therapeutic approaches. Given the onset of lung disease in early childhood, and the predicted further increase in life expectancy of patients with CF in the era of CFTR-directed therapeutics (3–5), avoidance of potential unwanted side effects of imaging techniques is increasingly important. In this context, despite its broad availability, the life-long use of chest computed tomography (CT) is limited by the risks associated with the cumulative dose of radiation exposure (6, 7). Over the past 15 years, magnetic resonance imaging (MRI) has emerged as an alternative to detect lung abnormalities in CF. (8–11) Advantages of MRI compared to CT are the absence of potentially harmful ionizing radiation, as well as the ability to perform simultaneous functional imaging studies for a combined assessment of lung morphology and functional parameters such as lung perfusion (12–16).

Morpho-functional MRI is an established technique that combines spatially resolved sequences to detect morphological changes and temporally resolved first-pass lung perfusion imaging with i.v. application of a contrast agent to assess abnormalities in lung perfusion. For semi-quantitative assessment of CF lung disease a morpho-functional chest MRI score was established that is based on a visual scoring system consisting of five morphological subscores including bronchiectasis/wall thickening and airway mucus plugging, and a perfusion score (17). This MRI score was shown to be robust and reproducible in single-and multicenter observational studies and to be sensitive to detect lung abnormalities across a broad age range of clinically stable patients with CF from infancy to adulthood (10, 11, 18), acute changes associated with pulmonary exacerbations (9), and response to therapeutic interventions including antibiotic therapy for pulmonary exacerbation, as well as cystic fibrosis transmembrane conductance regulator (CFTR) modulator therapies (13, 19, 20). However, first-pass perfusion imaging uses gadolinium-containing intravenous contrast agents that are generally safe and well tolerated, but also have some disadvantages. Gadolinium-based contrast agents can cause intolerance reactions, and although not observed with current macrocyclic contrast agents, previously used linear contrast agents were found to cause cerebral gadolinium deposits (21–23). Therefore, avoidance of intravenous contrast agents is desirable for long-term patient safety. Additional advantages of contrast agent-free MRI include shortening of the examination time, cost savings, no need for venipuncture that can be particularly stressful for children, and no contamination of the environment and groundwater by excreted contrast medium. Therefore, contrast agent-free MRI provides an important and patient-relevant technical advance for sensitive monitoring of CF lung disease.

Several techniques exist to derive information about lung perfusion from contrast agent-free MRI (24–29). Fourier decomposition MRI (FD-MRI) (30) was the basis for several derivatives. Matrix pencil decomposition MRI (MP-MRI) (31, 32) and phase resolved functional lung MRI (PREFUL-MRI) (29) have been shown to simultaneously assess lung ventilation and perfusion in CF patients (16, 33, 34). Previously, simultaneous assessment of lung ventilation and perfusion was only possible with nuclear medicine techniques with the use of radioisotopes, whereas inhalation of hyperpolarized gases only allows studies of ventilation (12, 35–37). Another advantage of MP-MRI is the possibility to evaluate data not only visually, but also by automated software analysis. This may open a possibility to replace contrast-enhanced (CE) perfusion MRI with a contrast agent-free technique.

The aim of this study was to investigate the correlation between visually scored and automatically calculated MP-MRI parameters for lung perfusion and ventilation with the morpho-functional MRI score as well as lung function testing. We therefore conducted a prospective observational study in 39 patients with CF who underwent morpho-functional MRI, including contrast-enhanced perfusion MRI, and MP-MRI as part of the same examination and assessment of lung function by spirometry.

## Materials and methods

### Study design and participants

This prospective observational study was approved by the Ethics Committee of Charité-Universitätsmedizin Berlin (EA2/170/20) and informed written consent was obtained from all patients or their legal guardians. During a two-year period, a total of 47 CF patients aged eight years and older in stable clinical condition were prospectively recruited for this study. In seven patients, the patients or their legal guardians refused intravenous contrast injection and in one case CE perfusion MRI was not evaluable due to a previously unknown thrombosis of the subclavian vein (which caused delayed inflow of the contrast agent). This led to exclusion of eight patients from the analysis and a study population of 39 patients.

MRI and spirometry were performed as simultaneously as possible, which meant the same day for 20 of 39 patients. The maximum interval between the two examinations was 14 days (mean: 1 day). Patient characteristics are summarized in Table 1.

**Table 1:** Characteristics of study population.

Characteristic	Mean ± SD
Number of patients	39
Age, in years (range)	21.6 ± 10.7 (8-45)
Male/female (%)	18/21 (46.2/53.8)
Weight, in kg	52.8 ± 16.9
Height, in cm	161.0 ± 16.6
BMI (kg/m^2^)	19.7 ± 3.6
*CFTR genotype*	
F508del/F508del (%)	19 (48.7)
F808del/other (%)	14 (35.9)
Other/other (%)	6 (15.4)
*CFTR modulator therapy*	
Lum-Iva (%)	8 (20.5)
Tez-Iva (%)	2 (5.1)
Elx-Tez-Iva (%)	3 (7.7)
None	26 (66.7)
Pancreatic insufficiency (%)	34 (87.2)
CF-related diabetes (%)	6 (15.4)
Chronic *Pseudomonas aeruginosa* (%)	9 (23.1)
Chronic NTM (%)	0 (0)

BMI, body mass index; CFTR, cystic fibrosis transmembrane conductance regulator; Elx, elexacaftor; Iva, ivacaftor; Lum, lumacaftor; Tez, tezacaftor; NTM, nontuberculous mycobacteria. Data are presented as mean ± SD, if not indicated otherwise.

### Magnetic resonance imaging

All imaging was acquired with the same 1.5 Tesla whole-body MR scanner (MAGNETOM Aera, Siemens Healthineers AG, Erlangen, Germany). MRI, including morphological sequences, MP-MRI and CE perfusion imaging, was always performed in the same examination and in the same order. Various versions of the examination protocol for mucoobstructive diseases exist at our center, in which technical parameters such as the field of view have been adapted to different age groups. The total examination time for the complete protocol averaged 15–20 min.

Morpho-functional MRI was performed as described previously (10, 11, 17), using a protocol comprising a balanced steady-state free-precession sequence (TrueFISP, Siemens) in axial, coronal and sagittal planes, T1-weighted spoiled gradient echo sequences (VIBE, Siemens) in axial and coronal planes before and after i.v. contrast administration and a fat-saturated T2-weighted sequence with rotating phase encoding (BLADE, Siemens) in axial and coronal planes (10, 11, 13, 15, 38). First-pass lung perfusion images were acquired with a spoiled gradient-echo sequence (time resolved angiography with stochastic trajectories, TWIST, Siemens) in coronal plane. During acquisition of this sequence a contrast bolus was injected via a peripheral catheter or central line at a rate of 2–4 mL/s and the first passage of the contrast bolus through the pulmonary circulation was captured within a dynamic series of up to 30 volume datasets of the whole lung, each with an acquisition time of 1.5 s. Two macrocycle-structured gadolinium-based contrast agents approved for this indication, Gadobutrol (Gadovist^©^, Bayer, Germany) and Gadoterate meglumine (Dotarem^©^, Guerbet, France), were used equally in the study. The amount of contrast medium was adjusted to the weight of our patients and was 1.5–8 mL gadobutrol, respectively, 2.3–16 mL gadoterate meglumine. As gadobutrol is twice as concentrated (1.0 mmoL/mL) as gadoterate meglumine (0.5 mmoL/mL), the total injection volume was only half as high in each case. Perfusion image datasets were post-processed by subtracting the baseline images (acquired prior to the contrast agent administration) from those with maximal contrast enhancement in the lung parenchyma.

Contrast agent-free functional imaging was performed as previously described using the MP-MRI technique (30–32, 39, 40). MP-MRI is a derivative of Fourier decomposition MRI and a non-invasive and contrast agent-free technique for the assessment of pulmonary ventilation and perfusion (32). MP-MRI relies on dynamic free-breathing ultra-fast balanced steady-state free precession (uf-bSSFP) acquisitions of chest images and provides regional ventilation and perfusion information from a single acquisition series (39). The uf-bSSFP pulse sequence uses an optimized excitation pulses and gradient switching patterns of a conventional Cartesian bSSFP imaging scheme accompanied by partial echo readouts and ramp sampling techniques. As a result, echo time and repetition time are shortened, which improves signal in the lung parenchyma and reduces motion as well as off-resonance artifacts. In order to cover the whole chest volume, imaging is performed using between 8–10 slices. Each slice consists of 160 coronal images acquired in approximately 50 s. Spectral information extracted using MP decomposition comprising respiratory and cardiac frequencies, amplitudes and phases is used to generate spatially-resolved fractional ventilation and perfusion maps. The technique requires no further patient compliance such as prolonged breath-holding. The time-resolved uf-bSSFP image series are processed by elastic image registration for compensation of respiratory motion (40). The registration algorithm preserves signal magnitude in each image but automatically aligns lung structures such as vessels, thoracic wall and airways. Subsequently, the lung regions on the registered images are automatically segmented using an artificial neural network (31). The motion-corrected and segmented images are further analyzed voxel-wise using matrix pencil decomposition to extract the amplitudes of the respiratory and cardiac signal modulations in the lung parenchyma, and were used to generate fractional ventilation and perfusion images. The procedures for image registration, lung segmentation using an artificial neural network and matrix pencil decomposition were combined into an image processing pipeline for MP-MRI. The software was written in Python and C++ for Linux operating system using CUDA Toolkit 12.0 (NVIDIA Corp., Santa Clara, CA), ITK library 5.2 (41) and Armadillo library 12.0 (NICTA, Brisbane, Australia). The image postprocessing and analysis was performed on a workstation equipped with 2x Epyc 7502 CPU (AMD Inc. Santa Clara, CA) and Quatro RTX 8000 GPU (NVIDIA Corp.). The data processing took approximately 10 min per subject. MP-MRI was always performed before contrast agent injection to avoid bias caused by the contrast agent.

### Visual image assessment

In the first step of data analysis, two experienced thoracic radiologists with 13 years (MOW) and 6 years (FD) of experience in chest MRI of CF patients were blinded to clinical and demographic characteristics and applied the principles of the validated morpho-functional MRI score (17) to morphological MRI sequences, subtraction images of contrast-enhanced first-pass perfusion imaging and to color-coded maps computed from MP-MRI data.

**Figure 1:**
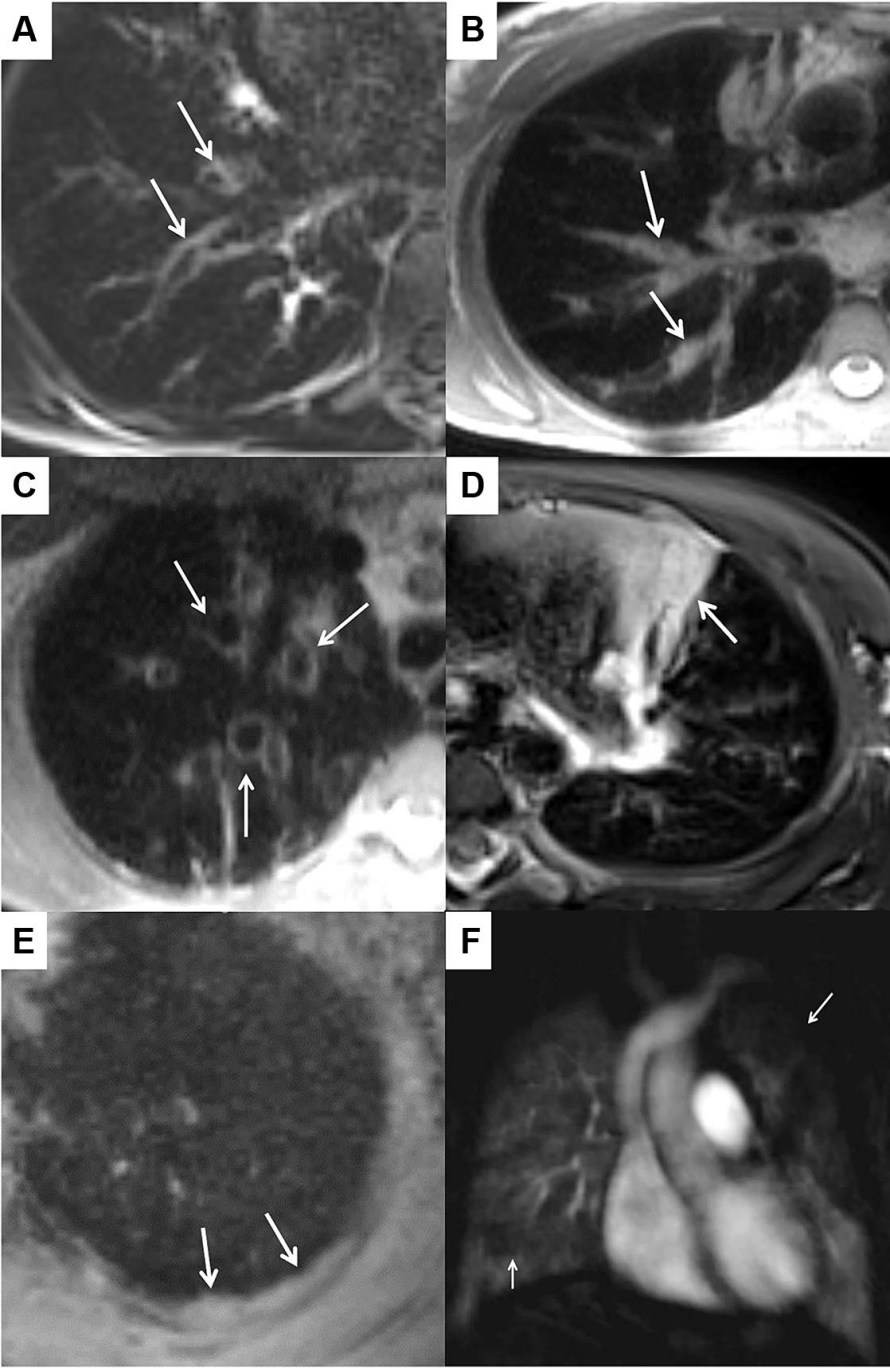
CF-related MRI findings representing the subscores of the morpho-functional chest MRI score. **(A–E)** Show image sections of a high-resolution T2-weighted sequence acquired in transversal plane. The arrows indicate bronchiectasis/wall thickening **(A)**, mucus plugging **(B)**, sacculations **(C)**, consolidation **(D)** and an inflammatory pleural thickening **(E)**, which is part of the score as a “special finding.” **(F)** Shows a post-processed subtraction image from the contrast-enhanced perfusion MRI acquired in coronal plane. The arrows mark a large perfusion deficit in the left upper lobe and a small perfusion deficit in the middle lobe. By definition, each subscore in each lobe is assessed individually and 0–2 points are scored per lobe depending on whether the corresponding pathology is not present (0), involves less than 50% (1) or more than 50% (2) of the lobe.

In the morpho-functional MRI score, five morphologic subscores and a perfusion score assessed on subtracted perfusion maps from contrast-enhanced first-pass perfusion imaging are assessed for each lobe and the lingula separately. The five morphologic subscores for (I) bronchiectasis and wall thickening, (II) mucus plugging, (III) sacculations and abscesses, (IV) consolidations, and (V) pleural reactions (including effusions) sum up to the morphology score and the global score results from the sum of morphology score and perfusion score (17). Figure 1 shows CF-related MRI findings resulting in the morpho-functional chest MRI score. The extent of pulmonary morphologic and perfusion abnormalities is graded separately for each lobe as follows: 0 (no abnormality), 1 (<50% of the lobe), or 2 (≥50% of lobe affected). Because this system considers the lingula as a separate, sixth lobe, maximum scores of 12 can be obtained for each subscore, 60 for the morphology score, and 72 for the global score.

The color-coded MP-MRI perfusion and ventilation maps were scored in analogy to the MRI perfusion score, but blinded to morpho-functional MRI (including CE perfusion MRI) and the results of automated software analysis. The principle of the morpho-functional score was easily transferred to the color-coded visualizations of the MP-MRI perfusion and ventilation analyses, which were also scored with 0–1–2 points for each lobe separately.

The scores obtained by the two readers were very consistent and data were analysed using arithmetic means.

### Automated assessment of MP-MRI data

In the second step of data analysis, fractional ventilation and perfusion MP-MRI data were automatically analyzed using a dedicated in-house developed software (31). The distributions of voxel values from ventilation and perfusion images obtained in the segmented lung regions ( S ) were used to calculate a threshold value ( T ) used to identify regions with ventilation and perfusion impairment. Lung regions with values below the threshold value were classified as regions with functional impairment. The threshold values were calculated for each slice independently. The extent of perfusion and ventilation defects detected by MP-MRI was expressed as the percentage of hypoperfused lung volume (perfusion defect percentage, QDP) and hypoventilated lung volume (ventilation defect percentage, VDP) to the total lung volume. In addition, an experimental parameter “ventilation/perfusion overlap” (VQO) was calculated, describing the spatial overlap of ventilation and perfusion. The parameter is expressed as the percentage of simultanously hypoperfused and hypoventilated lung volume. VQO can be interpreted as a measure of the hypoxic pulmonary vasoconstriction in which the localized hypoxia leads to vasoconstriction with reduced perfusion in the affected bronchovascular territory.

Instead of using a median-based threshold as in previous studies with MP-MRI, we employed a method which estimates the threshold from the mean difference between maximum and minimum intensity projections applied on the ventilation and perfusion images. The main advantage of this method is its improved robustness to signal inhomogeneities caused by coil sensitivities. Threshold values ( T ) used for the identification of impaired ventilation and perfusion lung regions were calculated is the following way:

- Maximum and minimum intensity projections were performed for voxels inside the two-dimensional segmented lung region (*S*) along each dimension:
$${MIP}_{x}\left(y\right)=\max\left(S\left(x,y\right)\right),{MIP}_{y}\left(x\right)=\max\left(S\left(x,y\right)\right)$$

and

$${\mathrm{MinIP}}_{x}\left(y\right)=\min\left(S\left(x,y\right)\right),{\mathrm{MinIP}}_{y}\left(x\right)=\min\left(S\left(x,y\right)\right)$$

- The resulting one-dimensional projections were then interpolated to the same maximum number of elements for calculation of the square-root of the Hadamard product:
$${MIP}_{xy}=\sqrt{{MIP}_{x}\cdot {MIP}_{y}},{\mathrm{MinIP}}_{xy}=\sqrt{{\mathrm{MinIP}}_{x}\cdot {\mathrm{MinIP}}_{y}}$$
- The threshold T is calculated as the mean value of the resulting maximum and minimum projection difference:
$$T=\bar{{MIP}_{xy}-{\mathrm{MinIP}}_{xy}}$$

Visual and software analysis of MP-MRI data was possible in all patients. MP-MRI postprocessing was fully automated and required no manual interaction.

### Spirometry

Spirometry was performed according to standards endorsed by the American Thoracic Society and European Respiratory Society (42, 43). Percent predicted values were calculated using the Global Lung Initiative (GLI) reference values (44). Forced expiratory volume in one second (FEV1), mid-expiratory flow at 25% of vital capacity (MEF25) and forced vital capacity (FVC) were included in the analyses. Percent predicted results were based on equations of the global lung function initiative (45).

## Statistical analysis

Statistical analyses were performed with R version 4.0.2 (R Foundation for Statistical Computing, Vienna, Austria). According to histograms and Kolmogorov–Smirnov tests, a normal distribution of metric data was not assumed. Data are presented as mean ± SD, median, interquartile range (IQR; 25th–75th percentile), and range (min–max). Spearman’s rank correlation coefficient *r* was used to describe the association between metric parameters. The correlation coefficient *r* measures how close the relationship between two parameters is to linearity. As two parameters can correlate both positively and negatively, *r* can be a minimum of −1.0 and a maximum of 1.0 (46). According to *Rowntree* and *Karlik* the correlations are assumed to be negligible (*r* = 0.0–0.2), weak/low (*r* = 0.2–0.4), moderate (*r* = 0.4–0.7), strong (0.7–0.9), and very strong (0.9–1.0) (46, 47). A *p*-value <0.05 was considered statistically significant.

## Results

### Characteristics of study population

**Figure 2:**
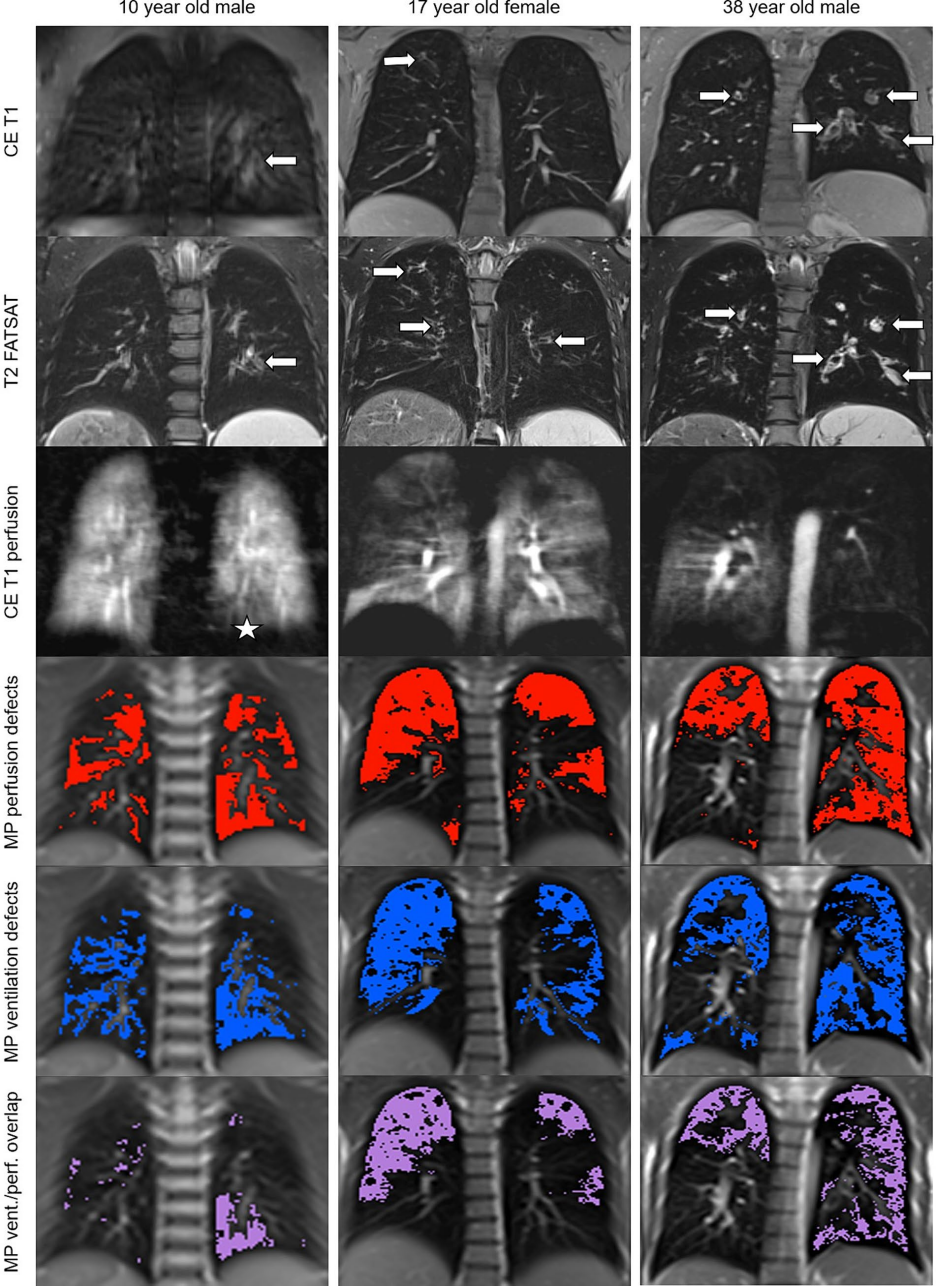
Changes in lung morphology, perfusion and ventilation detected by morpho-functional MRI and MP-MRI in patients with CF. Representative images of a 10 year old school child, a 17 year old adolescent and a 38 year old adult with CF. Contrast-enhanced T1-weighted and fat-saturated T2-weighted MRI shows dilated and/or wall-thickened bronchi (arrows). Contrast-enhanced perfusion MRI and color-coded visualizations of MP-MRI data show areas of decreased lung perfusion and ventilation as well as the overlap between the perfusion and ventilation defects that match with the location of the above morphological changes (asterisk in the youngest patient, obvious in the other two).

Thirty-nine patients with CF aged 8 to 45 years with a mean age of 21.6 ± 10.7 years and a mean percent predicted forced expiratory volume in 1 s (ppFEV1) of 76 ± 28% were enrolled in this study. Our study population showed an almost balanced sex ratio and the distribution of CFTR genotypes and proportion of exocrine pancreatic insufficiency was representative for CF patients in Central Europe (Table 1). Nine out of 39 patients were chronically infected with *Pseudomonas aeruginosa*, and one-third of the patients was on CFTR modulator therapy at the time of the study MRI (Table 1). MRI detected signs of CF lung disease in all patients (Figure 2 and Table 2). As previously reported (10, 11), the subscore bronchiectasis/wall thickening was the most important contributor to the MRI morphology score, followed by the mucus plugging subscore, whereas the contribution of the other morphologic subscores (i.e., abscesses/sacculations; consolidation; special findings) was low in our cohort of clinically stable patients (Tables 2, 3). The MRI global score was 24.5 ± 9.9 and the CE perfusion score was 6.9 ± 2.8. Visual scores of the experimental MP-MRI sequences were 7.2 ± 1.7 for the MP perfusion score and 6.7 ± 1.8 for the MP ventilation score (Table 3).

**Table 2:** Prevalence and magnitude of changes in lung structure and perfusion detected by chest MRI in patients with CF in the total study population and grouped by age.

Age group, years		Total	≤9	10–19	20–29	30–39	≥40
Patients, *n*		39	4	16	9	7	3
Mean age, years		21.6	8.5	13.8	24.8	33.6	42.7
Mean BMI		19.7	14.4	19.2	21.1	21.6	21.6
Global	Prevalence	39 (100)	4 (100)	16 (100)	9 (100)	7 (100)	3 (100)
	Score	24.5 (9.9)	21.1 (6.3)	16.9 (6.9)	29.4 (8.3)	34.6 (5.2)	31.2 (3.1)
Morphology	Prevalence	39 (100)	4 (100)	16 (100)	9 (100)	7 (100)	3 (100)
	Score	17.6 (7.4)	15.6 (5.5)	12.0 (5.6)	21.0 (5.7)	25.0 (4.2)	22.3 (2.8)
Bronchiectasis/wall thickening	Prevalence	39 (100)	4 (100)	16 (100)	9 (100)	7 (100)	3 (100)
	Score	8.7 (2.6)	8.1 (2.1)	6.6 (1.6)	9.7 (2.3)	11.4 (1.1)	10.8 (1.1)
Mucus plugging	Prevalence	39 (100)	4 (100)	16 (100)	9 (100)	7 (100)	3 (100)
	Score	4.9 (2.5)	4.4 (0.7)	3.1 (1.5)	6.8 (2.5)	5.9 (2.3)	7.2 (1.5)
Abscesses/sacculations	Prevalence	13 (33.3)	0 (0)	3 (18.8)	3 (33.3)	5 (71.4)	2 (66.7)
	Score	0.4 (0.9)	0.0 (0.0)	0.2 (0.4)	0.6 (1.4)	1.0 (0.8)	0.3 (0.2)
Consolidation	Prevalence	25 (64.1)	1 (25.0)	7 (43.8)	7 (77.8)	7 (85.7)	3 (100)
	Score	1.0 (1.3)	0.3 (0.4)	0.7 (1.1)	0.9 (0.7)	2.5 (1.6)	1.0 (0.4)
Special findings	Prevalence	32 (82.1)	3 (75.0)	10 (62.5)	9 (100)	7 (100)	3 (100)
	Score	2.6 (2.1)	2.9 (2.4)	1.5 (1.6)	3.1 (1.7)	4.3 (1.8)	3.0 (1.5)
CE perfusion	Prevalence	39 (100)	4 (100)	16 (100)	9 (100)	7 (100)	3 (100)
	Score	6.9 (2.8)	5.5 (1.2)	4.8 (1.8)	8.4 (2.8)	9.6 (1.3)	8.8 (1.0)
MP perfusion	Prevalence	39 (100)	4 (100)	16 (100)	9 (100)	7 (100)	3 (100)
	Score	7.2 (1.7)	7.0 (0.9)	6.0 (1.2)	7.9 (1.7)	8.7 (1.2)	7.5 (0.0)
MP ventilation	Prevalence	39 (100)	4 (100)	16 (100)	9 (100)	7 (100)	3 (100)
	Score	6.7 (1.8)	6.8 (0.3)	5.5 (1.4)	7.2 (1.8)	8.6 (1.3)	6.7 (0.6)

Prevalence data are presented as *n* of patients (percentage) and absolute MRI scores as mean (SD). BMI, body mass index; CE, contrast-enhanced; MP-MRI, matrix pencil decomposition MRI; MRI, magnetic resonance imaging.

**Table 3:** Results of spirometry, morpho-functional MRI and MP-MRI.

	Parameters	Mean	SD	Median	Q25	Q75	Min	Max	Range
PFT	ppFEV1	76.1	28.3	78.0	51.6	100.3	28.1	127.5	99.4
	ppMEF25	47.8	36.7	37.3	18.4	72.7	6.6	139.3	132.7
	ppFVC	87.7	21.1	89.0	73.8	105.7	42.4	132.7	90.3
Morpho-functional MRI	Global score	24.5	9.9	27.5	14.8	31.2	7.5	42.0	34.5
	Morphology score	17.6	7.4	20.0	10.0	23.5	5.0	32.5	27.5
	Bronchiectasis/wall thickening	8.7	2.6	9.0	6.0	11.2	4.0	12.0	8.0
	Mucus plugging	4.9	2.5	4.5	3.0	6.0	0.5	11.0	10.5
	Abscesses/sacculations	0.4	0.9	0.0	0.0	0.5	0.0	4.5	4.5
	Consolidation	1.0	1.3	0.5	0.0	1.2	0.0	4.0	4.0
	Special findings	2.6	2.1	2.0	1.0	4.2	0.0	7.0	7.0
	CE perfusion score	6.9	2.8	6.5	5.0	9.0	0.5	11.5	11.0
MP-MRI	MP perfusion score	7.2	1.7	7.0	6.0	8.5	3.5	10.5	7.0
	MP ventilation score	6.7	1.8	6.5	5.5	7.2	2.5	10.5	8.0
	QDP	22.8	19.7	45.3	29.0	60.9	1.4	74.0	72.6
	VDP	44.5	15.3	19.5	10.9	35.2	0.3	53.7	53.4
	VQO	38.7	18.5	39.0	23.3	54.9	4.0	69.8	65.8

CE, contrast-enhanced; MP, matrix pencil decomposition; MRI, magnetic resonance imaging; PFT, pulmonary function test (spirometry); ppFEV1, percent predicted forced expiratory volume in 1 s; ppFVC, percent predicted forced vital capacity; ppMEF25, percent predicted maximum expiratory flow at 25 percent; Q25, 25th quartile; Q75, 75th quartile; QDP, ventilation defect percentage; SD, standard deviation; VDP, perfusion defect percentage; VQO, ventilation/perfusion overlap.

### MP-MRI enables assessment of lung perfusion and correlates with contrast-enhanced perfusion MRI

**Figure 3:**
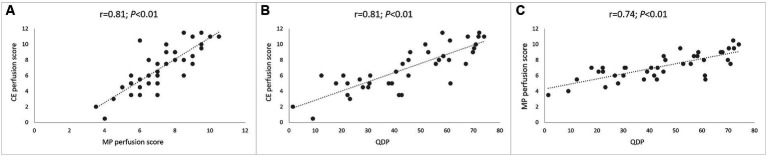
Correlation between lung perfusion determined by MP-MRI and contrast-enhanced perfusion MRI. **(A)** Correlation between visual MP perfusion score and visual CE perfusion score. **(B)** Correlation between automated QDP and visual CE perfusion score. **(C)** Correlation between automated QDP and visual MP perfusion score. Spearman’s rank correlation coefficient *r* and *p*-values are provided for each correlation.

**Figure 4:**
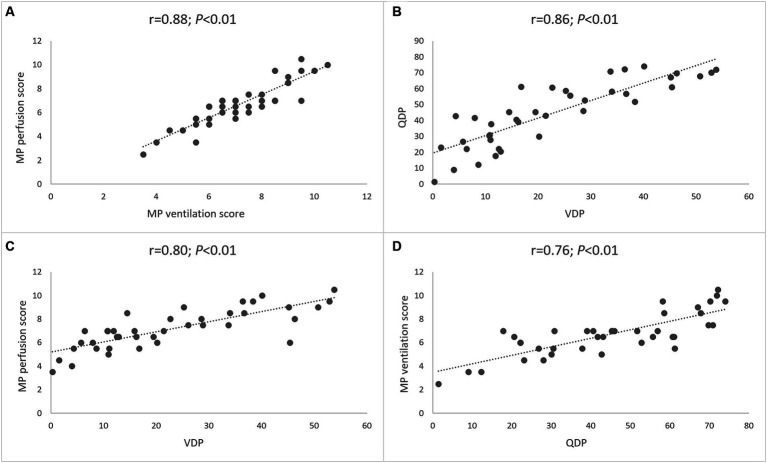
Correlation between morpho-functional MRI scores and MP-MRI parameters. The first and second row shows correlations of the MRI global score and visual MP perfusion score **(A)**, automated perfusion defect percentage **(B)**, visual MP ventilation score **(C)** and automated ventilation defect percentage **(D)**. The third and fourth row shows correlations of the MRI morphology score and visual MP perfusion score **(E)**, automated perfusion defect percentage **(F)**, visual MP ventilation score **(G)** and automated ventilation defect percentage **(H)**. Spearman’s rank correlation coefficient *r* and *p*-values are provided for each correlation.

First, we compared assessment of lung perfusion by MP-MRI to CE first-pass MR angiography as the established gold standard for studies of lung perfusion by MRI (Figure 2). Visual evaluation of the MP perfusion score showed a strong correlation with the established CE perfusion score (*r* = 0.81; *p* < 0.01) (Figure 3). Similarly, QDP derived from automated analysis of MP-MRI showed a strong correlation with the CE perfusion score (*r* = 0.81; *p* < 0.01). Comparison of the two MP-MRI parameters of lung perfusion showed a strong correlation between the visual MP perfusion score and automated QDP (*r* = 0.74; *p* < 0.01) (Figure 4 ). MP perfusion score and QDP also showed strong correlations with MRI global score, MRI morphology score, and bronchiectasis/wall thickening subscore as well as the CE perfusion score (Figure 4 and Table 4).

**Table 4:** Correlations of spirometry, morpho-functional MRI and MP-MRI (Spearman’s rank correlation coefficient *r*, all *p*-values <0.01).

		PFT			Morpho-functional MRI								MP-MRI				
		ppFEV1	ppMEF 25	ppFVC	Global score	Morphology score	Bronchiectasis/wall thickening	Mucus plugging	Abscesses/sacculations	Consolidation	Special findings	CE perfusion score	MP perfusion score	MP ventilation score	QDP	VDP	VQO
PFT	ppFEV1		0.95	0.92	−0.76	−0.70	−0.71	−0.56	−0.42	−0.39	−0.54	−0.79	−0.69	−0.75	−0.78	−0.83	−0.84
	ppMEF 25	0.95		0.83	−0.78	−0.72	−0.74	−0.58	−0.51	−0.44	−0.57	−0.80	−0.68	−0.70	−0.75	−0.82	−0.74
	ppFVC	0.92	0.83		−0.63	−0.54	−0.58	−0.44	−0.25	−0.24	−0.38	−0.68	−0.62	−0.67	−0.73	−0.74	−0.77
Morpho-functional MRI	Global score	−0.76	−0.78	−0.63		0.98	0.88	0.83	0.59	0.71	0.81	0.93	0.78	0.76	0.80	0.83	0.80
	Morphology score	−0.70	−0.72	−0.54	0.98		0.86	0.83	0.61	0.77	0.84	0.85	0.72	0.68	0.74	0.73	0.74
	Bronchiectasis/wall thickening	−0.71	−0.74	−0.58	0.88	0.86		0.78	0.47	0.58	0.65	0.83	0.80	0.70	0.73	0.79	0.76
	Mucus plugging	−0.56	−0.58	−0.44	0.83	0.83	0.78		0.31	0.57	0.58	0.74	0.68	0.58	0.64	0.70	0.66
	Abscesses/sacculations	−0.42	−0.51	−0.25	0.59	0.61	0.47	0.31		0.65	0.59	0.52	0.33	0.37	0.33	0.32	0.34
	Consolidation	−0.39	−0.44	−0.24	0.71	0.77	0.58	0.57	0.65		0.67	0.55	0.37	0.26	0.41	0.39	0.36
	Special findings	−0.54	−0.57	−0.38	0.81	0.84	0.65	0.58	0.59	0.67		0.74	0.55	0.57	0.57	0.51	0.53
	CE perfusion score	−0.79	−0.80	−0.68	0.93	0.85	0.83	0.74	0.52	0.55	0.74		0.81	0.80	0.81	0.89	0.87
MP-MRI	MP perfusion score	−0.69	−0.68	−0.62	0.78	0.72	0.80	0.68	0.33	0.37	0.55	0.81		0.88	0.74	0.80	0.81
	MP ventilation score	−0.75	−0.70	−0.67	0.76	0.68	0.70	0.58	0.37	0.26	0.57	0.80	0.88		0.76	0.78	0.79
	QDP	−0.78	−0.75	−0.65	0.80	0.74	0.73	0.64	0.33	0.41	0.57	0.81	0.74	0.76		0.86	0.94
	VDP	−0.83	−0.82	−0.74	0.83	0.73	0.79	0.70	0.32	0.39	0.51	0.89	0.80	0.78	0.86		0.95
	VQO	−0.84	−0.74	−0.77	0.80	0.74	0.76	0.66	0.34	0.36	0.53	0.87	0.81	0.79	0.94	0.95	

CE, contrast-enhanced; FVC, forced vital capacity; MEF 25, maximum expiratory flow at 25 percent; MP, matrix pencil decomposition; MRI, magnetic resonance imaging; PFT, pulmonary function test (spirometry); ppFEV1, percent predicted forced expiratory volume in 1 s; QDP, ventilation defect percentage; VDP, perfusion defect percentage; VQO, ventilation/perfusion overlap.

### MP-MRI enables assessment of pulmonary ventilation and correlates with spirometry

**Figure 5:**
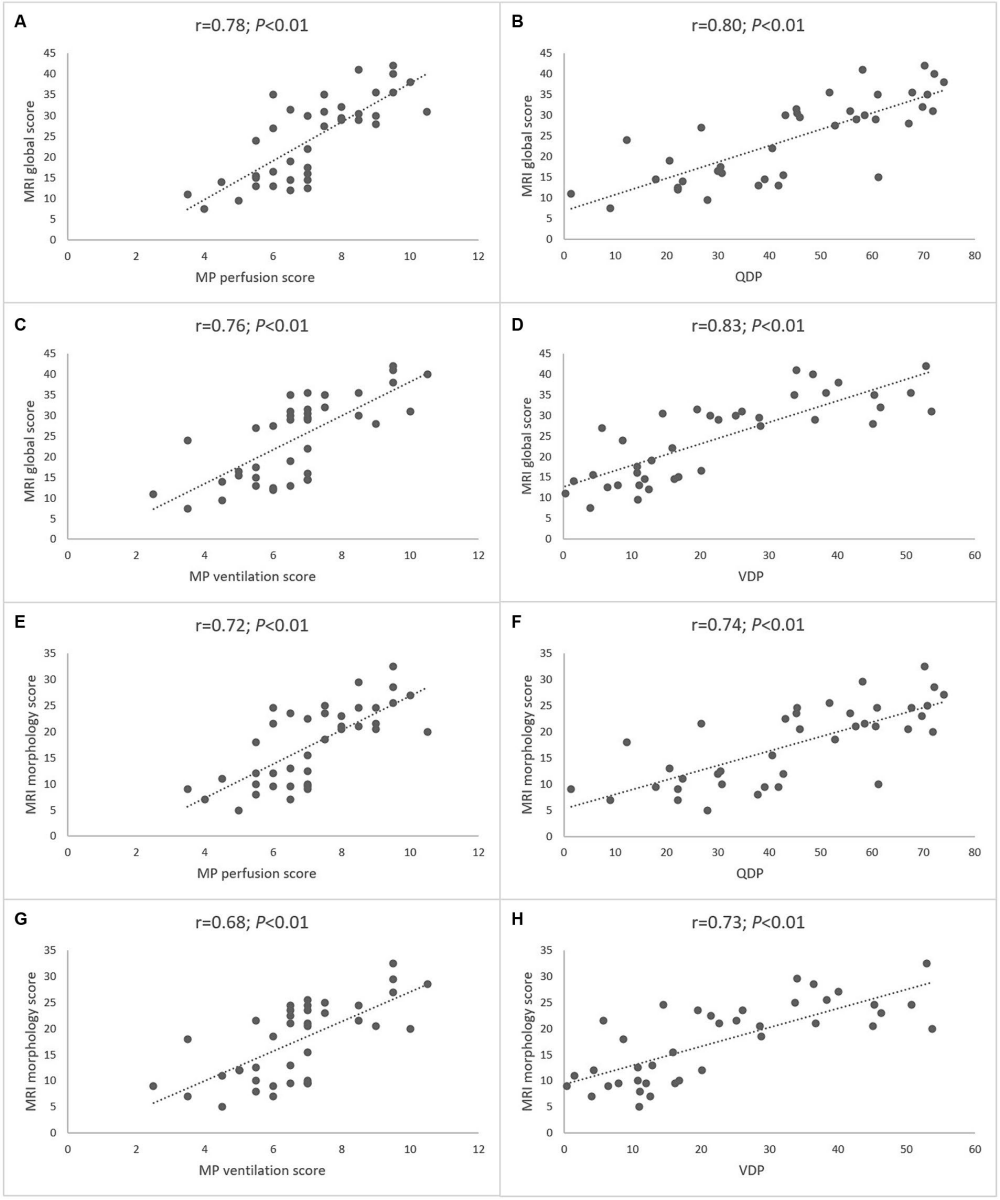
Correlation between lung ventilation determined by MP-MRI and spirometry. **(A)** Correlation between visual MP ventilation score and ppFEV1. **(B)** Correlation between automated VDP and ppFEV1. **(C)** Correlation between automated VDP and visual MP ventilation score. Spearman’s rank correlation coefficient *r* and *p*-values are provided for each correlation.

Next, we determined the relationship between MP-MRI ventilation and spirometry. The visual MP ventilation score showed strong inverse correlations with ppFEV1 (*r* = −0.75; *p* < 0.01) and other spirometry outcomes (Figure 5 and Table 4). Further, VDP determined from automated MP-MRI analysis showed a strong inverse correlation with ppFEV1 (*r* = −0.83; *p* < 0.01) and other spirometry outcomes (Figure 5 and Table 4). The visual MP ventilation score and automated VDP were strongly correlated with each other (*r* = 0.78; *p* < 0.01) (Figure 5). Both MP ventilation parameters, the MP ventilation score and VDP, showed strong correlations with the MRI global score, MRI morphology score and bronchiectasis/wall thickening subscore as well as the CE perfusion score (Figure 4 and Table 4).

### Relationship between lung perfusion and ventilation determined by MP-MRI

**Figure 6:**
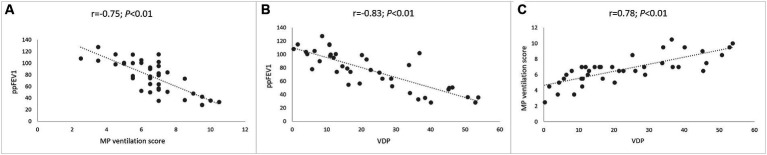
Relationship between lung perfusion and ventilation determined by MP-MRI. **(A)** Correlation between visual MP ventilation score and MP perfusion score. **(B)** Correlation between automated VDP and QDP. **(C)** Correlation between automated VDP and visual MP perfusion score. **(D)** Correlation between automated QDP and visual MP ventilation score. Spearman’s rank correlation coefficient *r* and *p*-values are provided for each correlation.

Finally, we determined the relationship between lung perfusion and ventilation determined by MP-MRI in CF lung disease. The visual MP perfusion score showed a strong correlation with the MP ventilation score (*r* = 0.88; *p* < 0.01) (Figure 6). Similarly, the automated QDP showed a strong correlation with VDP (*r* = 0.86; *p* < 0.01) (Figure 6). Finally, a crossover comparison showed intermediate to strong correlations between the visual MP perfusion score and VDP, and between the visual MP ventilation score and QDP (Figure 6). The experimental parameter ventilation/perfusion overlap (VQO) showed comparably strong correlations with the spirometry results and the visual scores as the more established parameters QDP and VDP from previous studies (Table 4). Taken together, these results demonstrate the capability of MP-MRI for simultaneous quantitative assessment of abnormalities in lung ventilation and perfusion by MRI in our cohort of CF patients.

## Discussion

In a cohort of 39 CF patients with a broad range of lung disease severity, we show that lung perfusion determined by contrast agent-free MP-MRI correlates strongly with CE perfusion MRI (2, 10, 11, 28, 48). Second, we show that MP-MRI enables simultaneous quantitative assessment of lung perfusion and ventilation, and that lung ventilation determined by MP-MRI shows a strong correlation with spirometry. Third, our MP-MRI-derived metrics of lung perfusion and ventilation demonstrate a strong cross-correlation in patients with CF. Finally, we show that automated analysis of MP-MRI computing QDP and VDP strongly correlates with reader-based visual scoring of MP-MRI and may therefore be used for unbiased quantitative analysis of changes in lung perfusion and ventilation. Collectively, our data support the use of MP-MRI for non-invasive quantitative studies of abnormalities in lung perfusion and ventilation in patients with CF.

To the best of our knowledge, this is the first study that directly compared contrast agent-free functional MP-MRI, established contrast-enhanced morpho-functional MRI, and spirometry for detection of abnormalities in the lungs of patients with CF. It is also the first study to use MP-MRI in adult CF patients. However, this is not the first study ever to use contrast-agent-free functional MP-MRI in patients with cystic fibrosis. In a study from 2018, 23 CF patients aged 6–18 years and a comparison group of healthy children underwent MP-MRI on two consecutive days (49). The automated post-processing of the MRI data was carried out with a previous version of the software used in our study. QDP and VDP were almost identical in both examinations, which proved a very good short-term reproducibility of the MP-MRI measurements. In addition, QDP and VDP were significantly higher in the CF patients than in the healthy controls. As in our study, a strong correlation was found between FEV1 and VDP (*r* = 0.65), but this was even stronger in our study (*r* = 0.83). In another, more recent study, 24 children with CF were examined with MP-MRI and spirometry at one-year intervals in order to demonstrate the therapeutic effect of CFTR modulator therapy (16). Morphologic lung changes were assessed visually using the chest MRI score as in our study and MP-MRI data were quantified using a previous version of our software. The positive therapeutic effect was demonstrated by the fact that QDP and VDP decreased significantly under modulator therapy. However, both MP-MRI studies on CF patients listed here were performed without contrast administration, which prevented a comparison of the MP-MRI perfusion analyses with the gold standard of MRI perfusion imaging.

Abnormalities in lung perfusion are a hallmark of CF that are associated with lung disease severity in pediatric and adult patients (10, 11, 13, 17). The MP-MRI technique used for contrast agent-free assessment of lung perfusion in our study is a further development of FD-MRI initially introduced more than a decade ago (30). The strong correlation between perfusion signals obtained by MP-MRI and CE MRI, i.e., the current standard of reference for MRI-based perfusion studies, support the future use of MP-MRI for contrast agent-free quantification of perfusion defects associated with CF lung disease. Another derivative of FD-MRI is perfusion-weighted phase-resolved functional lung (PREFUL) MRI (29), that has also been used successfully for contrast agent-free assessment of lung perfusion in CF, chronic obstructive pulmonary disease (COPD) and chronic thrombembolic pulmonary hypertension (CTEPH) (34, 50–52). Similar to the visual perfusion scores we used in the present study, binary perfusion defect maps were generated and semi-quantitatively evaluated to compare the perfusion signals obtained with PREFUL-MRI to CE perfusion MRI and single-photon emission computed tomography SPECT (50). While a good overall agreement between the perfusion maps across the three different techniques was reported, this decreased significantly near the heart and diaphragm in the maps derived from PREFUL-MRI data, probably due to susceptibility to motion artifacts (50). Such a decrease in agreement between FD-based perfusion signals and CE perfusion MRI in the aforementioned artifact-prone regions was not observed for the MP-MRI perfusion maps generated in our study (Figure 2), suggesting a potential advantage for assessment of abnormal perfusion in these lung regions.

Another advantage of MRI is that it can be used for the assessment of lung ventilation (2, 27, 32, 49, 51, 52). So far, the most common approach for MRI-based ventilation imaging has been the use of hyperpolarized gases such as ^3^He, ^129^Xe and ^19^F as inhaled contrast agents that enable visualization of regional ventilation defects in patients with CF. (53–56) However, with a very limited number of sites equipped to perform hyperpolarized gas MRI worldwide, access to this technique remains limited, also limiting its use for multi-center studies. FD-MRI-based techniques such as MP-MRI allow assessment of pulmonary ventilation in addition to pulmonary perfusion and can be installed on standard 1.5 T MRI scanners. This facilitates their use for diagnostic monitoring as well as outcome measurement in multicenter studies, as previously shown for contrast-enhanced morpho-functional MRI (10, 13, 18, 20). For application at higher field strength (i.e., 3 T) data acquisition with spoiled gradient echo (SPGR) based techniques or transient SPGR pulse sequence can be better suited as the mitigation of the off-resonance artifacts with bSSFP imaging used by MP-MRI is more demanding at high magnetic field strength (57). However, the clinical experience with MP-MRI at 3 T is currently limited.

The strong correlation between ventilation determined by MP-MRI and spirometry observed in our study supports a potential role of MP-MRI for quantitative, but also regional assessment of lung function in patients with CF. Similarly, PREFUL-MRI was used successfully for assessment of lung ventilation in patients with chronic pulmonary diseases including CF. (14, 51, 52, 58) The ultra-fast balanced steady-state free precession sequence of the MP-MRI technique has the advantage that lung perfusion and ventilation can be assessed simultaneously, leading to an examination time of 6–7 min for complete acquisition of MP-MRI data in free breathing, whereas slightly longer examination time has been reported in studies using PREFUL-MRI (14). On the other hand, there are obvious advantages of PREFUL-MRI over MP-MRI. The most important is the significantly better portability of this contrast-agent-free functional MRI technique to MRI scanners from other manufacturers and with other field strengths, since PREFUL-MRI is now a commercially available protocol and MP-MRI uses a custom uf-bSSFP pulse sequence—although MP-MRI is now part of the routine at our center. However, a direct comparison of MP-MRI with PREFUL-MRI for contrast agent-free functional lung imaging is still pending. The strong correlation between lung ventilation and perfusion found in our study is consistent with results from a previous experimental study that compared FD-MRI, CE perfusion MRI, and ^3^He MRI in a porcine model of artificial bronchus occlusion (35), as well as previous studies comparing the lung clearance index derived from the multiple-breath washout technique with contrast-enhanced perfusion derived from morpho-functional MRI in patients with CF. (10) Collectively, these results support that regional ventilation impairment due to airway mucus plugging leads to hypoxic pulmonary vasoconstriction as an important mechanism of impaired lung perfusion in CF.

MP-MRI uses data acquired during tidal breathing and employs image registration to compensate for the respiratory motion. Main outcome parameters characterizing the functional impairment, namely VDP and QDP, are generally robust with respect to respiratory patterns. The distribution of the perfusion signal used for calculation of QDP is spectrally isolated from other signal modulations caused by respiratory motion. The fractional ventilation depends on the breathing amplitude and the density change of lung parenchyma. VDP as a relative measure of ventilation inhomogeneity does not depend on the absolute values of fractional ventilation. However, low density changes in the lung tissue caused by shallow breathing can more difficult to detect due to the inherently low signal of the lung and corruption by noise.

This study has limitations. Our cross-sectional study in clinically stable patients does not provide information on the causal and long-term relationship between impairments in lung ventilation and perfusion detected by MP-MRI in CF lung disease. Therefore, longitudinal studies ideally including infants and preschool children will be required to address pertinent questions such as changes during acute pulmonary exacerbations and reversibility of abnormalities and response to therapy including CFTR modulator therapy that targets the underlying cause of the disease at different stages of CF lung disease. These longitudinal studies will be facilitated by the automated quantitative analysis of MP-MRI ventilation and perfusion data presented in our study. As the software available for this study was not yet able to calculate regional differences, the automated parameters QDP and VDP are currently global measurements without making use of the information on spatial distribution, i.e., similar to other outcome measures of lung function such as spirometry (10). However, the automated parameters QDP and VDP were strongly correlated with visual scores so that regional information may be obtained from the color-coded visualizations by reader-based scoring, and analysis software for automated analysis at regional resolution is expected to become available in the near future (31).

In summary, this study demonstrates for the first time that MP-MRI enables simultaneous assessment of lung perfusion and ventilation over a broad range of CF lung disease severity employing 1.5 T MRI without the need for intravenous or inhaled contrast agents. Together with the possibility of fully automated data analysis, these properties support MP-MRI as novel functional non-invasive quantitative outcome measure for diagnostic monitoring and clinical trials in patients with CF and potentially other muco-obstructive lung diseases in conjunction with morphological MRI.

## Data availability statement

The raw data supporting the conclusions of this article will be made available by the authors, without undue reservation.

## Ethics statement

The studies involving humans were approved by Ethikkommission der Charité Universitätsmedizin Berlin. The studies were conducted in accordance with the local legislation and institutional requirements. Written informed consent for participation in this study was provided by the participants’ legal guardians/next of kin.

## Author contributions

FD: Conceptualization, Data curation, Formal analysis, Investigation, Methodology, Validation, Visualization, Writing – original draft, Writing – review & editing. GB: Conceptualization, Data curation, Formal analysis, Investigation, Methodology, Software, Supervision, Validation, Visualization, Writing – original draft, Writing – review & editing. JR: Data curation, Formal analysis, Investigation, Writing – review & editing. MS: Supervision, Validation, Writing – review & editing, Conceptualization, Investigation, Methodology. HP: Data curation, Formal analysis, Investigation, Writing – review & editing. IS: Conceptualization, Data curation, Formal analysis, Investigation, Software, Supervision, Validation, Writing – original draft, Writing – review & editing. OP: Data curation, Formal analysis, Investigation, Software, Writing – review & editing. OB: Investigation, Project administration, Software, Supervision, Writing – review & editing. MW: Conceptualization, Data curation, Formal analysis, Investigation, Methodology, Supervision, Validation, Writing – review & editing. MM: Conceptualization, Formal analysis, Funding acquisition, Investigation, Methodology, Project administration, Resources, Supervision, Validation, Writing – review & editing.
